# The influence of race/ethnicity and place of service on breast reconstruction for Medicare beneficiaries with mastectomy

**DOI:** 10.1186/2193-1801-3-416

**Published:** 2014-08-08

**Authors:** Tracy Onega, Julie Weiss, Karla Kerlikowske, Karen Wernli, Diana SM Buist, Louise M Henderson, Martha Goodrich, Jennifer Alford-Teaster, Beth Virnig, Anna NA Tosteson, Wendy DeMartini, Rebecca Hubbard

**Affiliations:** Department of Community & Family Medicine, Geisel School of Medicine at Dartmouth, HB 7927 Rubin 8, Lebanon, NH 03756 USA; Norris Cotton Cancer Center, Geisel School of Medicine at Dartmouth, Lebanon, NH USA; The Dartmouth Institute for Health Policy and Clinical Practice, Geisel School of Medicine at Dartmouth, Lebanon, NH USA; Departments of Medicine and Epidemiology and Biostatistics, University of California, San Francisco, CA USA; General Internal Medicine Section, Department of Veterans Affairs, University of California, San Francisco, CA USA; Group Health Research Institute, Seattle, WA USA; Department of Radiology, The University of North Carolina, Chapel Hill, NC USA; School of Public Health, University of Minnesota, Minneapolis, MN USA; Department of Radiology, University of Wisconsin School of Medicine and Public Health, Madison, WI 53792-3252 USA

**Keywords:** Reconstruction, Disparities, Mastectomy, Breast cancer

## Abstract

Racial disparities in breast reconstruction for breast cancer are documented. Place of service has contributed to disparities in cancer care; but the interaction of race/ethnicity and place of service has not been explicitly examined. We examined whether place of service modified the effect of race/ethnicity on receipt of reconstruction.

We included women with a mastectomy for incident breast cancer in SEER-Medicare from 2005–2009. Using Medicare claims, we determined breast reconstruction within 6 months. Facility characteristics included: rural/urban location, teaching status, NCI Cancer Center designation, cooperative oncology group membership, Disproportionate Share Hospital (DSH) status, and breast surgery volume. Using multivariable logistic regression, we analyzed reconstruction in relation to minority status and facility characteristics.

Of the 17,958 women, 14.2% were racial/ethnic women of color and a total of 9.3% had reconstruction. Caucasians disproportionately received care at non-teaching hospitals (53% v. 42%) and did not at Disproportionate Share Hospitals (77% v. 86%). Women of color had 55% lower odds of reconstruction than Caucasians (OR = 0.45; 95% CI 0.37-0.55). Those in lower median income areas had lower odds of receiving reconstruction, regardless of race/ethnicity. Odds of reconstruction reduced at rural, non-teaching and cooperative oncology group hospitals, and lower surgery volume facilities. Facility effects on odds of reconstruction were similar in analyses stratified by race/ethnicity status.

Race/ethnicity and facility characteristics have independent effects on utilization of breast reconstruction, with no significant interaction. This suggests that, regardless of a woman’s race/ethnicity, the place of service influences the likelihood of reconstruction.

## Introduction

Breast reconstruction following mastectomy for breast cancer is associated with better quality of life, (Alderman et al. 2006) lower decisional regret, improved self-image, and other benefits, relative to mastectomy without reconstruction (Albornoz et al. 2013). In fact, based on evidence and political will, the 1999 Women’s Health and Cancer Rights Act (WHCRA) mandated insurance coverage for breast reconstruction after mastectomy to try to ensure access (Albornoz et al. 2013). Despite the WHCRA, and additional legislation in 2001 that imposed penalties on insurers for non-coverage, reports of women of all ages eligible for breast reconstruction receiving it range from <20% to 60% (Jagsi et al. 2014; Alderman et al. 2009; Christian et al. 2006; Hershman et al. 2012). Women of color have been shown to be significantly less likely to receive breast reconstruction – for African American women, less than half as likely as Caucasian women (Alderman et al. 2006; Jagsi et al. 2014; Alderman et al. 2009; Alderman et al. 2003; Bian et al. 2008). The reasons underlying this disparity are not clear, although the disparity may relate to other factors shown to decrease the likelihood of breast reconstruction, such as marital status, rural residence, comorbidities, insurance, surgeon volume, hospital volume, hospital size, (Hershman et al. 2012) teaching hospital status, and cancer center designation (Kruper et al. 2011a).

Racial disparities may be due in part to where patients receive care (Morris et al. 2010). For example, a recent study of women with breast cancer showed that type of hospital at which women received definitive therapy explained some of the racial disparities observed in treatment (Keating et al. 2009). Our understanding of how race/ethnicity and place of service interact to affect utilization of care and outcomes for cancer is lacking, but is vital to understanding and developing interventions to reduce disparities. While both race/ethnicity and facility characteristics have been examined, no studies to date have explicitly examined the interaction of race/ethnicity and hospital characteristics on utilization of breast reconstruction. The objective of this study was to explicitly examine how Caucasian and women of color with breast cancer differ in their utilization of breast reconstruction following mastectomy and whether effects associated with the types of facilities they use vary for Caucasian and women of color.

## Methods

### Data

We used data from the National Cancer Institute’s Surveillance, Epidemiology, and End Results (SEER) program – linked to Medicare claims (National Cancer Institute 2014). At the time of these analyses, SEER captured incident cancer cases from 17 population-based registries, (2014) representing 26% of the U.S. population. SEER records standardized patient demographic, clinical, and vital status information in the Patient Entitlement and Diagnosis Summary File (PEDSF). Individuals within the SEER registries are linked to Medicare data from the time of eligibility until death through an algorithm with a 93% match rate (Warren et al. 2002). This study used Medicare beneficiary information (Denominator File), inpatient data (MedPAR), outpatient data (Outpatient Claims), and physician visit data (Carrier Claims). We also used the Hospital File, which links to both MedPAR and Outpatient claims files at the facility level to provide facility characteristics information based on the Healthcare Cost Report Information System (HCRIS) and the Provider of Service (POS) Survey administered through the Centers for Medicaid and Medicare Services (CMS) (2014).

### Study population

We included age-eligible women in Medicare with an incident breast cancer from 2005–2009 recorded in SEER (N = 114,454), with claims through 2010. We excluded women diagnosed upon autopsy (N = 1,619), those who did not have part A & B coverage (N = 13,174) or had HMO coverage (N = 28,468) one year before and 6 months after diagnosis. We further excluded women without pathologic confirmation of breast cancer (N = 786); reported from a nursing home (N = 10); reported surgery as not recommended (N = 3,029) or contraindicated (N = 205); who died prior to surgery (N = 1); or who had an unknown reason for surgery (N = 561), refused surgery (N = 434), or reported surgery as recommended but unknown if performed (N = 362). Finally, we then included the remaining women with early stage breast cancer (Stages 0, I, and II), non-missing race/ethnicity, mastectomy within 6 months of diagnosis, and who had a provider link to the Hospital File, resulting in an analytic sample of 17,958 women.

### Key variables – exposures, outcome, and covariates

The main exposures of interest in this study were race/ethnicity and characteristics of the facility at which a woman’s mastectomy occurred. Race/ethnicity was ascertained through the SEER PEDSF File, as 6 mutually- exclusive categories: Caucasian, African American, Hispanic, Asian, Native American, and Other. We further categorized race/ethnicity into: 1) women of color (African American, Hispanic, Asian, Native American, Other); and 2) Caucasian. Facility characteristics included: urban/rural, teaching hospital, NCI Cancer Center, cooperative oncology group member, and Disproportionate Share Hospital (DSH) – those that provide a certain amount of uncompensated care, (2014) as taken from the Hospital File linked to the mastectomy facility. In addition, we derived hospital-level breast cancer surgery volume empirically based on all mastectomy and breast conserving surgeries observed in claims from the study period attributed to unique facilities, and categorized into quartiles (surgery N -- Quartile 1: 1–8; Quartile 2: 9–18; Quartile 3: 19–35; Quartile 4: 36+).

Our outcome of interest was receipt of breast reconstruction following mastectomy (which occurred within 6 months of breast cancer diagnosis). We ascertained breast reconstruction from MedPAR and Outpatient Files using ICD-9 and CPT/HCPCS codes (Table 1). We identified breast reconstruction from the date of mastectomy and 6 months thereafter.

**Table 1:** **Procedure codes used in this analysis**

Procedure	ICD-9 Procedure Codes	CPT Codes	DRG Codes	Time Interval
**Mastectomy**	85.3, 85.33-85.36, 85.4, 85.41-85.48, V51.0	19180, 19182, 19200, 19240, 19303-19307	257, 258, 582, 583	Breast cancer diagnosis date + 6 months/Any mastectomy occurring during diagnosis years 2005-2009^α^
**Breast conserving surgery**	85.22, 85.23	19160, 19162, 19301, 19302	259, 260	Any BCS occurring during diagnosis years 2005-2009^α^
**Reconstruction**				
Implant	85.33, 85.35, 85.53, 85.54, 85.6, 85.89, 85.93-85.95	19340, 19342		Mastectomy + 6 months
Flap	85.7, 85.70-85.76, 85.79, 85.82-85.85, 85.87, 86.60, 86.70, 86.72, 86.74, 86.75			Mastectomy + 6 months
Tissue Expander	85.96	19357		Mastectomy + 6 months
Autologous		19350, 19361, 19364, 19366-19369		Mastectomy + 6 months

Abbreviations: CPT, current procedural terminology; ICD, International Classification of Diseases; DRG, Diagnosis Related Groups.

^α^Time frame for calculation of breast surgery volume.

We included covariates that we considered *a priori* to be potential confounders: age at diagnosis, urban/rural residence SEER registry, comorbidities, stage at diagnosis and quartile of median household income for census tract of residence at time of diagnosis. All of these covariates were taken from the PEDSF, except for comorbidities, which were derived from the claims file (MedPAR, Outpatient, and Carrier) using the Klabunde adaptation (Klabunde et al. 2000) of the Charlson Index (Charlson et al. 1987).

### Analyses

We conducted descriptive analyses of patient characteristics, and the characteristics of the hospitals at which their mastectomies were performed, overall and by receipt of reconstruction. We also stratified facility characteristics and median income by race/ethnicity to examine their joint distributions. Multivariable logistic regression models were used to estimate the odds of reconstruction in relation to women and facility characteristics. We developed an overall model for receipt of reconstruction, as well as a models stratified by Caucasian/women of color. To formally test for interactions between race/ethnicity and facility characteristics, fully adjusted regression models were run with each race/ethnicity and facility characteristic interaction. Statistical power for multivariable modeling was not adequate to separately model Hispanic, Asian, Native American, or Other racial/ethnic groups. We therefore modeled odds of reconstruction for Caucasian and women of color, including the facility characteristics: teaching hospital, urban/rural location, NCI Cancer Center, member of one or more cooperative oncology groups, DSH, and breast cancer surgery volume. Covariates included were: age in years (66–69, 70–74, 75–79, 80–84, 85+), quartile of income based on residence, rural/urban residence, SEER registry, stage at diagnosis, and comorbidities. Sensitivity analyses included: similar models but only with Caucasians and African Americans; and forward stepwise logistic regression models, observing changes in the race/ethnicity point estimate as facility characteristics were added to the multivariable model. All analyses were conducted using SAS 9.3 (Cary, NC) and a p-value < 0.05 was considered statistically significant.

## Results

Of the 17,958 women in the cohort, 85.8% were Caucasian, 8.3% were African American, and 6.0% were Asian, Native American, Hispanic, and Other race/ethnicity combined (Table 2). Women were distributed relatively evenly across five-year age groups up to 84 years, with a notably lower proportion of women over age 85 years (13.1%) (Table 2). The majority of women had no comorbidities (59.7%), lived in an urban location (86.1%), and had early stage invasive cancer (85.9%) (Table 2). Approximately half of the women attended a teaching hospital or a hospital belonging to one or more cooperative oncology groups, but only 3.7% attended an NCI Cancer Center (Table 2). The frequency of reconstruction was higher among: younger Medicare beneficiaries, Caucasians, women with an urban residence, higher income quartile in census tract of residence, those with DCIS, and no comorbidities (Table 2). Immediate reconstruction (same day as mastectomy) accounted for the vast majority (>90%) of cases, while the 99^th^ percentile for time to reconstruction was 160 days.

**Table 2:** **Reconstruction** ^**α**^ **among early stage breast cancer female Medicare beneficiaries with a mastectomy (N = 17,958) from 2005-2009**

	Without reconstruction	With reconstruction	Total
**Women's characteristics** ^**β**^	N (%)	N (%)	N (column%)
Total	16,297 (90.7)	1,661 (9.3)	17,958 (100.0)
Age (years)			
66-69	2,734 (16.8)	714 (43.0)	3,448 (19.2)
70-74	3,828 (23.5)	574 (34.6)	4,402 (24.5)
75-79	4,062 (24.9)	279 (16.8)	4,341 (24.2)
80-84	3,345 (20.5)	65 (3.9)	3,410 (19.0)
85+	2,328 (14.3)	29 (1.7)	2,357 (13.1)
Race/ethnicity			
Caucasian	13,882 (85.2)	1,522 (91.6)	15,404 (85.8)
African American	1,402 (8.6)	82 (4.9)	1,484 (8.3)
Other ^τ^	1,013 (6.2)	57 (3.4)	1,070 (6.0)
Urban/Rural			
Urban	13,909 (85.4)	1,545 (93.0)	15,454 (86.1)
Rural	2,385 (14.6)	116 (7.0)	2,501 (13.9)
Median income			
Quartile 4 (> = $59,450)	3,731 (23.1)	717 (43.4)	4,448 (25.0)
Quartile 3 ($44,000-$59,449)	4,020 (24.9)	441 (26.7)	4,461 (25.1)
Quartile 2 ($33,750-43,999)	4,147 (25.7)	283 (17.1)	4,430 (24.9)
Quartile 1 (<$33,750)	4,239 (26.3)	213 (12.9)	4,452 (25.0)
Stage at diagnosis			
0	2,331 (14.3)	382 (23.0)	2,713 (15.1)
I	6,959 (42.7)	730 (44.0)	7,689 (42.8)
II	7,007 (43.0)	549 (33.0)	7,556 (42.1)
Comorbidities			
0	9,504 (58.3)	1,217 (73.3)	10,721 (59.7)
1	4,204 (25.8)	335 (20.2)	4,539 (25.3)
2+	2,589 (15.9)	109 (6.6)	2,698 (15.0)
**Facility characteristics** ^**β**^			
Urban/Rural			
Urban	13,733 (86.0)	1,596 (97.7)	15,329 (87.1)
Rural	2,239 (14.0)	37 (2.3)	2,276 (12.9)
Teaching hospital			
No	8,516 (52.5)	671 (40.6)	9,187 (51.4)
Yes	7,710 (47.5)	981 (59.4)	8,691 (48.6)
NCI cancer center			
No	15,765 (96.7)	1,536 (92.5)	17,301 (96.3)
Yes	532 (3.3)	125 (7.5)	657 (3.7)
Cooperative oncology group			
No	8,146 (50.0)	535 (32.2)	8,681 (48.3)
Yes	8,151 (50.0)	1,126 (67.8)	9,277 (51.7)
Disproportionate share hospital			
No	3,503 (21.7)	448 (27.1)	3,951 (22.2)
Yes	12,634 (78.3)	1,203 (72.9)	13,837 (77.8)
Breast cancer surgery volume			
Quartile 4 (36+)	3,987 (24.6)	687 (41.5)	4,674 (26.1)
Quartile 3 (19-35)	4,129 (25.5)	444 (26.8)	4,576 (25.6)
Quartile 2 (9-18)	4,296 (26.5)	314 (19.0)	4,610 (25.8)
Quartile 1 (1-8)	3,815 (23.5)	210 (12.7)	4,025 (22.5)

^α^Reconstruction within 6 months of mastectomy.

^β^Missing (N): Women's Urban/Rural (3); Median Income (167); Facility's Urban/Rural (353); Teaching Hospital (80); Disproportionate Share Hospital (170); Breast Cancer Surgery Volume (76).

^τ^Other includes Hispanic, Asian, Native American and Other.

Examining facility characteristics stratified by racial/ethnic groups, African American women were more likely to receive mastectomies at teaching hospitals, and have more representation in hospitals with DSH designation than were other racial/ethnic groups (Table 3). Hispanic and Native American women were the least represented at facilities belonging to one or more cooperative oncology groups and to those with the highest quartile of breast cancer surgery volume (data not shown).

**Table 3:** **Women's census tract median income and facility characteristics by race/ethnicity among early stage breast cancer female Medicare beneficiaries with a mastectomy (N = 17,958) from 2005-2009**

	Caucasian (N = 15,404)	African American (N = 1,484)	Other ^β^(N = 1,070)
**Women's characteristics**	N (column%)		
Median income			
Quartile 4 (> = $59,450)	3,953 (25.9)	134 (9.1)	361 (34.0)
Quartile 3 ($44,000-$59,449)	3,962 (26.0)	221 (15.0)	278 (26.2)
Quartile 2 ($33,750-43,999)	3,917 (25.7)	306 (20.7)	207 (19.5)
Quartile 1 (<$33,750)	3,422 (22.4)	815 (55.2)	215 (20.3)
**Facility characteristics** ^**α**^			
Urban/Rural			
Urban	13,038 (86.4)	1,303 (89.3)	988 (94.1)
Rural	2058 (13.6)	156 (10.7)	62 (5.9)
Teaching hospital			
No	8,115 (52.9)	574 (38.8)	498 (46.9)
Yes	7,223 (47.1)	905 (61.2)	563 (53.1)
NCI cancer center			
No	14,858 (96.5)	1,433 (96.6)	1,010 (94.4)
Yes	546 (3.5)	51 (3.4)	60 (5.6)
Cooperative oncology group			
No	7,386 (48.0)	759 (51.2)	536 (50.1)
Yes	8,018 (52.0)	725 (48.8)	534 (49.9)
Disproportionate share hospital			
No	3,629 (23.8)	173 (11.7)	149 (14.1)
Yes	11,620 (76.2)	1,306 (88.3)	911 (85.9)
Breast cancer surgery volume			
Quartile 4 (36+)	4,070 (26.5)	376 (25.4)	228 (21.4)
Quartile 3 (19-35)	4,009 (26.1)	323 (21.8)	241 (22.7)
Quartile 2 (9-18)	3,930 (25.6)	393 (26.6)	287 (27.0)
Quartile 1 (1-8)	3,329 (21.7)	388 (26.2)	308 (29.0)

^α^Missing (N): Median Income (167); Facility's Urban/Rural (353); Teaching Hospital (80); Disproportionate Share Hospital (170); Breast Cancer Surgery Volume (76).

^β^Other includes Hispanic, Asian, Native American and Other.

**Figure 1:**
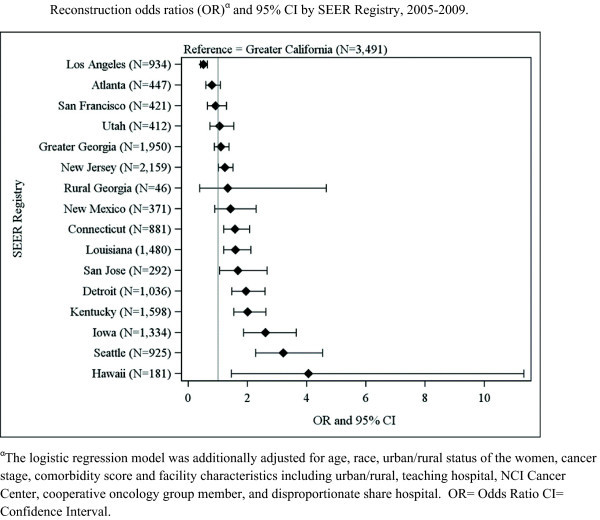
**Reconstruction odds ratios (OR)** ^**α**^ **and 95% CI by SEER Registry, 2005-2009.**

In multivariable logistic regression models, odds of breast reconstruction following mastectomy was significantly lower for women of color compared to Caucasian women (OR = 0.45; 95% CI 0.37-0.55), as well as for lower quartiles of census tract-level median household income, after adjusting for facility characteristics, SEER registry, age, rural/urban status, cancer stage, and comorbidity score (Table 4). Odds of reconstruction varied widely among the 16 SEER registries compared to the Greater California SEER registry ranging from more likely to receive reconstruction in Hawaii (OR = 4.07; 95% CI 1.46-11.33) to less likely in Los Angeles (OR = 0.51; 95% CI 0.41-0.64) (Figure 1). Several facility characteristics were also associated with lower odds of breast reconstruction, including: rural (OR = 0.33; 95% CI 0.23-0.48 relative to urban), a non-teaching hospital (OR = 0.75; 95% CI 0.65-0.85), not a cooperative oncology group member (OR = 0.68; 95% CI 0.59-0.77), and lower breast cancer surgery volume (Table 4). Estimates for facility characteristics were similar for Caucasian and women of color, in the stratified models, indicating that race/ethnicity does not modify associations between facility characteristics or median household income and receipt of reconstruction. No statistically significant interactions between race/ethnicity and any of the facility characteristics or median income were found when interaction terms for women of color status or median income with each facility characteristics were entered into the model. The characteristics of facility rurality and not being a member of a cooperative oncology group were strongly associated with non-receipt of reconstruction overall and similar results were found among Caucasian women and women of color (Table 4). Breast cancer surgery volume was not found to be statistically significant among the women of color (p = 0.41).

**Table 4:** **Odds ratios** ^**α**^ **and 95% confidence intervals for reconstruction within 6 months of a mastectomy among female Medicare beneficiaries with early stage breast cancer from 2005-2009 overall and stratified by race/ethnicity**

	Reconstruction within 6 Months		
	All Women	Caucasian	Women of color ^β^
**Women's characteristics**		OR (95% CI)^α^	
Race/ethnicity			
Caucasian (N = 15,404)	1.00 (referent)		
Women of color (N = 2,554)	0.45 (0.37-0.55)		
Median income	p for trend <0.0001	p for trend <0.0001	p for trend =0.08
Quartile 4 (> = $59,450)	1.00 (referent)	1.00 (referent)	1.00 (referent)
Quartile 3 ($44,000-$59,449)	0.73 (0.63-0.84)	0.72 (0.62-0.84)	0.78 (0.47-1.29)
Quartile 2 ($33,750-43,999)	0.54 (0.45-0.64)	0.53 (0.44-0.64)	0.52 (0.30-0.91)
Quartile 1 (<$33,750)	0.45 (0.37-0.55)	0.45 (0.36-0.56)	0.39 (0.22-0.69)
**Facility characteristics**			
Urban/Rural			
Urban	1.00 (referent)	1.00 (referent)	1.00 (referent)
Rural	0.33 (0.23-0.48)	0.34 (0.23-0.49)	0.24 (0.05-1.09)
Teaching hospital			
No	0.75 (0.66-0.85)	0.74 (0.65-0.85)	0.76 (0.49-1.17)
Yes	1.00 (referent)	1.00 (referent)	1.00 (referent)
NCI cancer center			
No	0.86 (0.68-1.09)	0.83 (0.65-1.06)	1.42 (0.64-3.16)
Yes	1.00 (referent)	1.00 (referent)	1.00 (referent)
Cooperative oncology group			
No	0.68 (0.59-0.77)	0.69 (0.60-0.79)	0.57 (0.36-0.90)
Yes	1.00 (referent)	1.00 (referent)	1.00 (referent)
Disproportionate share hospital			
No	1.00 (referent)	1.00 (referent)	1.00 (referent)
Yes	0.89 (0.78-1.02)	0.90 (0.78-1.04)	0.75 (0.45-1.27)
Breast cancer surgery volume	p for trend <0.0001	p for trend <0.0001	p for trend =0.41
Quartile 4 (36+)	1.00 (referent)	1.00 (referent)	1.00 (referent)
Quartile 3 (19-35)	0.76 (0.66-0.87)	0.75 (0.64-0.87)	0.89 (0.55-1.43)
Quartile 2 (9-18)	0.69 (0.59-0.81)	0.70 (0.59-0.84)	0.63 (0.36-1.11)
Quartile 1 (1-8)	0.73 (0.60-0.89)	0.72 (0.58-0.89)	0.89 (0.50-1.59)

^α^The logistic regression model was additionally adjusted for age, SEER registry, urban/rural status of the women, cancer stage, and comorbidity score. OR = Odds Ratio CI = Confidence Interval.

^β^Women of color includes African American, Hispanic, Asian, Native American and Other.

## Discussion

This study explicitly examined the joint effect of race/ethnicity and place of service on receipt of breast reconstruction. We found significant associations between receipt of breast reconstruction following mastectomy and race/ethnicity and place of service; with women of color and those at rural facilities, those with lower breast cancer surgery volume, not a cooperative oncology group member and non-teaching hospitals, less likely to receive reconstruction. We did not find an interaction between race/ethnicity and place of service on likelihood of reconstruction, suggesting that racial disparities persist despite place of service. We found some evidence of differential use of facility types for mastectomy by race/ethnicity, particularly higher proportions of women of color attending facilities that were not members of cooperative oncology groups, and with lower overall breast cancer surgery volumes. More women of color were in the lower area-level income strata than Caucasian, although in adjusted models, women of color were over half as likely to receive breast reconstruction as Caucasian. Significant regional variation based on SEER registry was also noted for breast reconstruction.

Our results add to the growing evidence around disparities in use of breast reconstruction following mastectomy. The procedure has state and federally-mandated coverage due to its documented benefits to quality of life, body image, self-esteem, and sexuality, (Albornoz et al. 2013) yet is disproportionately under-utilized by women of color and by some types of facilities. Several studies have shown similar decreased use of breast reconstruction for women of color (Alderman et al. 2006; Jagsi et al. 2014; Alderman et al. 2009; Alderman et al. 2003; Kruper et al. 2011a; Kruper et al. 2011b; Yang et al. 2013; Sisco et al. 2012). Prior studies have also demonstrated that facility characteristics have a significant impact on use of reconstruction following mastectomy, including: a) academic or teaching hospital; (Sisco et al. 2012; Reuben et al. 2009) b) urban hospital; (Reuben et al. 2009) and c) hospital volume and size (Hershman et al. 2012). Interestingly, in a study conducted in the National Comprehensive Cancer Network, race/ethnicity was not a significant determinant of breast reconstruction, (Christian et al. 2006) unlike the work presented here, in which racial/ethnic disparities persisted after controlling for facility characteristics.

The idea that racial/ethnic disparities in healthcare utilization and outcomes are at least partially explained by place of service is not novel. The Institute of Medicine (IOM) reviewed a large number of studies, both cancer and non-cancer related, and reported that racial/ethnic disparities remain, but are diminished, when accounting for some health care system attributes that influence access to and quality of care (Nelson 2002). Other work points to the influence of geographic location – and hence access to specific facilities and services – as an explanatory factor for some racial disparities in health care utilization (Baicker et al. 2005; Chandra et al. 2013; Skinner et al. 2003). The roles of location and facility characteristics in health care disparities are still not well understood, and even less so for cancer care specifically.

Because cancer care can be highly specialized, understanding facility characteristics that are associated with receipt of recommended and high-quality care is particularly important to either help replicate best practices in other facilities, or establish referral networks that facilitate equitable access. Several studies focused on cancer care have linked facility characteristics to recommended utilization and/or better outcomes, including higher volumes, teaching or academic medical centers, NCI Cancer Centers, and subspecialist providers. Some of this work further demonstrated that racial disparities in cancer outcomes (mortality) diminished when evaluating the effect of race within similar facilities (Onega et al. 2010). This suggests that reducing disparities in outcomes for cancer patients of all races/ethnicities will require a focus on improving access to high-quality cancer care.

Several limitations of this study should be noted. First, breast reconstruction is an elective procedure, which some women may not choose. Important factors that were not measured are likely to play a role, such as patient preference, cultural attitudes, and individual financial resources. Also, we used SEER-Medicare data, thus limiting our sample to women over age 65. We were also limited in sample size so were unable to analyze Hispanic, Asian, and Native American women separately. Finally, all women in the cohort had Medicare, so we could not observe the effect of insurance on reconstruction.

Understanding the components that create racial disparities in cancer care is vital to better redressing the problem. The evidence is beginning to move beyond simply identifying disparities, towards explaining them. One model of cancer disparities recognizes the role of factors along a hierarchical spectrum: biological/genotypic – individual – health care system -- social/neighborhood. Such a complex, multilevel model provides an important framework, and ultimately should be elucidated. This research sought to isolate the contribution of individual-level and health care system level factors on significantly lower use of breast reconstruction among cancer patients of color. The effects of place of service on breast reconstruction are significant, but similar for all women regardless of race/ethnicity. However, even when accounting for place of service, racial/ethnic disparities in breast reconstruction persist. Understanding which factors contribute to those disparities – and whether they are economic, cultural, behavioral, etc., is important in order to unravel the complexities of racial/ethnic disparities, and begin to identify the target for interventions.

### Ethical standards

All procedures were compliant with the Health Insurance Portability and Accountability Act (HIPAA) and approved by the Geisel School of Medicine’s Committee of Human Subjects Internal Review Board.
